# Enhancing medical education in respiratory diseases: efficacy of a 3D printing, problem-based, and case-based learning approach

**DOI:** 10.1186/s12909-023-04508-6

**Published:** 2023-07-17

**Authors:** Xuebo Yan, Yingying Zhu, Lei Fang, Peishan Ding, Shu Fang, Jinhua Zhou, Jiong Wang

**Affiliations:** 1 Department of Geriatric Respiratory and Critical Care, Institute of Respiratory Disease, Provincial Key Laboratory of Molecular Medicine for Geriatric disease, The First Affiliated Hospital of Anhui Medical University, 218 Jixi Road, Hefei, 230022 Anhui China; 2 School of Biomedical Engineering, Anhui Medical University, 81 Meishan Road, Hefei, 230023 Anhui China

**Keywords:** 3D printing technology, PBL teaching, CBL teaching, Respiratory disease, Clinical teaching

## Abstract

**Objectives:**

The present study aims to investigate the efficacy of utilizing three-dimensional (3D) printing technology in concert with Problem-Based Learning (PBL) and Case-Based Learning (CBL) pedagogical approaches in educating senior undergraduate clinical medical students on respiratory diseases.

**Methods:**

A cohort of 422 fourth-year clinical medicical students of from Anhui Medical University, pursuing a five-year program, were arbitrarily segregated into two distinct groups. The experimental group was subjected to a combined pedagogical approach, which included 3D printing technology, PBL and CBL (referred to as DPC). Conversely, the control group was exposed to conventional teaching methodologies for respiratory disease education. The effectiveness of the teaching methods was subsequently appraised using both theoretical test scores and custom questionnaires.

**Results:**

Post-quiz scores indicated a statistically significant improvement in the DPC group as compared to the traditional group (P < 0.01). Self-evaluation and satisfaction questionnaires revealed that the DPC group’s self-assessment scores outperformed the traditional group in several aspects, including clinical thinking ability, learning initiative, self-study ability, anatomical knowledge mastery, confidence in learning, ability to analyze and solve problems, comprehension of the knowledge, help to clinical thinking and level of satisfaction on the teaching methods (P < 0.01). However, within the unsatisfied DPC sub-group, none of these self-assessment aspects, except for comprehension of the knowledge, impacted the learning efficacy (P > 0.05).

**Conclusion:**

The deployment of the DPC pedagogical approach may confer unique experiential learning opportunities for students, potentially enhancing theoretical test scores and promoting self-evaluation and satisfaction in the context of respiratory disease education. Hence, it may be instrumental in augmenting the overall teaching efficacy.

**Supplementary Information:**

The online version contains supplementary material available at 10.1186/s12909-023-04508-6.

## Introduction

Over recent years, there has been a steady development and adoption of innovative and efficacious pedagogical methods in medical institutions. Among these advances, three-dimensional (3D) printing technology, characterized by the creation of physical objects through a layer-by-layer process based on digital blueprint files, has gained significant prominence. This widespread acceptance has been propelled by the emergence of economically viable desktop and personal 3D printers [1, 2]. Initially, 3D printing technology found significant relevance within the realm of medical imaging, however, it has since permeated other aspects of the medical field. Based on imaging data, this technology has fostered a plethora of applications, including surgical planning, implant and tissue design, medical research, and notably, in the sphere of medical education and training [1, 3]. The production of rapid prototyping objects through 3D printing has facilitated the creation of tangible models for medical education and simulation, proving instrumental in clinical training [2, 4]. The practice of clinical medicine necessitates an exhaustive understanding of human anatomy and an array of pertinent anatomical structures. Such knowledge has traditionally been imparted through traditional lectures and the dissection of human cadavers during the preclinical studies in medical school. However, two-dimensional representations on a computer screen may fail to provide a comprehensive and intuitive understanding of intricate anatomical intricacies crucial to patient procedures. Rapid prototyping ameliorates this limitation by enhancing the 3D learning experience, not just of standard anatomical structures, but also of pathological conditions [1]. Moreover, this approach paves the way for teaching and training of both generic and patient-specific surgical procedures, particularly in complex cases [3]. Rapid prototyping models enable intensive training of surgical procedures training, absolving the risk of patient complications [1, 3, 4]. The utilization of 3D printing technology extends to a myriad of clinical teaching contexts, including but not limited to, congenital heart surgery [5], cerebrovascular disease [6] and the administration of novel therapeutics in periodontal surgery [7], among others [8].

Problem-Based Learning (PBL) a pedagogical paradigm wherein learners are grouped under the aegis of non-directive tutors and tasked with real-world, or complex challenges reflecting situations that encompass genuine problems and experiences [9, 10]. As a learner-centric and interactive methodology, PBL stimulaes students to delve into such problems through self-study, research, discussion, and collaboration within their respective groups. Consequently, in contrast to traditional learning methods, PBL fosters independent problem-solving skills, promotes self-education, and cultivates sustainable learning abilities [9, 11]. A related educational paradigm within the medical academia is Case-Based Learning(CBL), whereby an instructor presents a case found on actual medical records, proffers questions and facilitates students in integrating disjointed concepts, articulating, analyzing, and ultimately, solving the problems under expert guidance [12]. Both PBL and CBL have proven instrumental within medical school curricula. A study by Elangovan et al. reported the integration of basic-clinical sciences, PBL, CBL, and Interprofessional Education (IPE) in U.S. dental schools’ curricula, thereby proposing an integrated curriculum model for the future [13]. The study incorporated 31 U.S. dental schools and revealed that while three-quarters of the participating schools continue to teach basic and clinical sciences independently, 61.3% reported having an integrated curriculum. Among the respondent schools, 16 had integrated a PBL component into their curricula (with two implementing PBL across all courses and 14 utilizing a hybrid PBL approach). Furthermore, two schools had incorporated CBL into all courses, while ten had integrated CBL into more than 75% of their courses [13. In the practical clinical instruction of thyroid disease [10, gastrointestinal tumor [14], dental education [15, etc. [12, a combined approach of PBL and CBL has proven effective and may be a promising mode for teaching.

The efficacy of the amalgamation of 3D printing technology, PBL and CBL in the domain of respiratory disease education remains relatively unexplored. The present study aims to evaluate the effectiveness of this composite approach, herein referred to as DPC (3D printing technology combined with PBL and CBL), specifically in the context of respiratory diseases such as bronchial lung cancer, one of the most prevalent respiratory diseases.

## Literature review

In the global panorama of continuous and exponential accrual of medical and clinical knowledge and technological advancements, the importance of rigorous and standardized medical education has been elevated [10]. Predicated on clinical knowledge and procedural skills, the capacity to perform medical case analysis has emerged as a pivotal aspect of training, supplementing on-the-spot response abilities and competencies [16]. Traditional lectures, while effective for large-scale dissemination of fundamental knowledge and concepts, continue to remain the most common instructional method. However, they demonstrate limited effectiveness in imparting crucial critical reasoning skills necessary for medical case analysis and on-the-spot response capabilities [17]. In the realm of respiratory clinical education, a multitude of diseases involving diverse structures such as the trachea, bronchus, lung lobe, lung segment and sub-segment, arteries, veins, lymph nodes, and interstitium exist [18]. As such, it is imperative for learners to possess a foundational understanding of anatomy prior to delving into the treatment of distinct types of respiratory diseases. Learners lacking practical experience are likely to encounter difficulties comprehending respiratory disease atlases, ultrasound images, and normal human lung models due to the inherent complexity in reconstructing anatomical structures [16, 18, 19]. Traditional classroom teaching methodologies are more suited to junior medical students who are primarily in the process of knowledge acquisition [20, 21]. For senior medical students, however, who are required to cultivate robust communication and clinical thinking skills, traditional approaches may prove sub-optimal [22]. Compared with traditional classroom teaching method, PBL and CBL have the potential to recreate a realistic medical environment. PBL fosters a group learning model that facilitates in-depth interaction between teachers and students, thereby enabling the achievement of personalized educational objectives [23]. Simultaneously, CBL, with its emphasis on teacher-guided instruction, aids students in forming more effective comprehensive clinical thinking habits through the provision of clinical case materials [24]. Given these individual strengths, our study proposes to amalgamate PBL and CBL in a bid to leverage their synergistic benefits.

Traditional pedagogical tools utilized for instruction in respiratory diseases often exhibit a relatively coarse texture, limited simulation capabilities, and lack diversity. These tools fall short of fulfilling the escalating educational requirements of advanced medical students. Recently, additive manufacturing technology, namely 3D printing, has been garnering significant attention due to its potential applications in a multitude of sectors, including healthcare [25]. This technology has found extensive use in a plethora of non-medical and medical fields [26–28]. Within the medical sphere, clinicians traditionally relied on two-dimensional X-ray images or two-dimensional images derived from computed tomography or magnetic resonance imaging for the three-dimensional reconstruction of lesions, thereby aiding in their comprehensive understanding [2, 29]. However, while such three-dimensional imaging enhances visualization of complex lesions, it lacks tactile characteristics. 3D printing technology, based on digital model files, employs a layer-by-layer printing technique to rapidly generate three-dimensional solid models. This process facilitates the creation of personalized, direct, and tactile models [30]. The resultant 3D printed model, which can be preserved for prolonged durations and reused, compensates for the limitations and deficiencies inherent in traditional teaching tools [31].

In summary, when compared to traditional instructional methods, PBL, CBL, and 3D printing each bring their unique advantages to the field of medical education. These innovative teaching methodologies have been successfully employed in the teaching of clinical nursing in areas such as congenital heart surgery [5], cerebrovascular disease [6], medical oncology [12], implant dentistry [32], thyroid disease [10], among others [7, 8, 14, 33]. In the present study, we aim to investigate the utility of an integrative approach combining 3D printing technology with PBL and CBL teaching methods (DPC) in the instruction of respiratory diseases.

## Methods

### Participants

We prospectively enrolled fourth-year students registered on the PBL study block as part of the BSc Degree in Clinical Medicine at Anhui Medical University from September 2021 to April 2022. They completed all the required respiratory disease courses that are provided at the Anhui Medical University. 422 fourth-year students of five-year clinical medicine from Anhui Medical University were divided into two groups. The participants were randomly divided into the “DPC group” featuring 3D printing technology combined with PBL and CBL teaching methods, or the “traditional group” featuring a lecture-based teaching program. The students were kept unaware of their group assignments prior to their internships. A simple randomization was adopted for this study [10]. Since the courses were arranged at different times, students and residents who took class at the same time were organized in ascending order by their student numbers. All students were renumbered as 1 to N [10]. If the assigned number was odd, he/she entered the DPC group, whereas if the number was even, he/she entered the traditional group. Each group was supervised by teaching staff consisting of one instructor and one assistant. Informed consent was obtained from all participants [10]. This study was approved by the Institutional Review Board and Ethics Committee of Anhui Medical University. Basic characteristics and information of participants were shown as Table 1. A total of 422 fourth-year students were enrolled. 211 students were assigned to the DPC group and other 211 students were assigned to the traditional group. In order to ensure that all students can attend class, if someone can’t attend this class temporarily, they can attend it with the students of the next class. The mean age of all the students was 21.73 ± 0.57. The mean age of the DPC group was 21.76 ± 0.55 and that of the traditional group was 21.70 ± 0.59. Among them, there were 214 male students, accounting for 50.71%, and 208 female students, accounting for 49.29%. In the DPC group, there were 109 male students, accounting for 51.66%, and 102 female students, accounting for 48.34%. In the traditional group, there were 105 male students, accounting for 49.76%, and 106 female students, accounting for 50.24%. In the DPC group, there were 24 students whose scores at last semester were arranged in the top 100, accounting for 11.37%. In the traditional group, there were 27 students whose scores at last semester were arranged in the top 100, accounting for 12.80%. There were no significant differences between the two groups in terms of gender, age, or proportion in the top 100 (P > 0.05).

**Table 1:** The basic characteristics of all the students

Item	DPC group [n (%)]	Traditional group [n (%)]	Statistics	P value
Gender			X^2^ = 0.15	0.70
Male	109 (51.66)	105 (49.76)		
Female	102 (48.34)	106 (50.24)		
Age	21.76 ± 0.55	21.70 ± 0.59	T = 0.97	0.33
Proportion				
Top 100	24 (11.37)	27 (12.80)	X^2^ = 0.20	0.65
Others	187 (88.63)	184 (87.20)		

### Study design

**Fig. 1:**
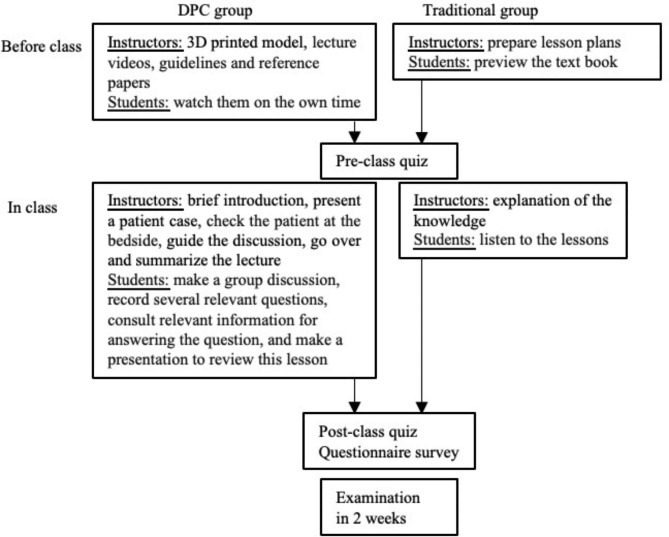
A flowchart of study design

We chose bronchial lung cancer as the topic for applying the 3D printing technology together with PBL and CBL teaching methods in this study because the diagnosis and treatment of bronchial lung cancer is one of the key courses that students must master in our department. A flowchart of the study design was shown as Fig. 1 with some modifications as descripted by Zhao et al. [10].

### DPC group

**Fig. 2:**
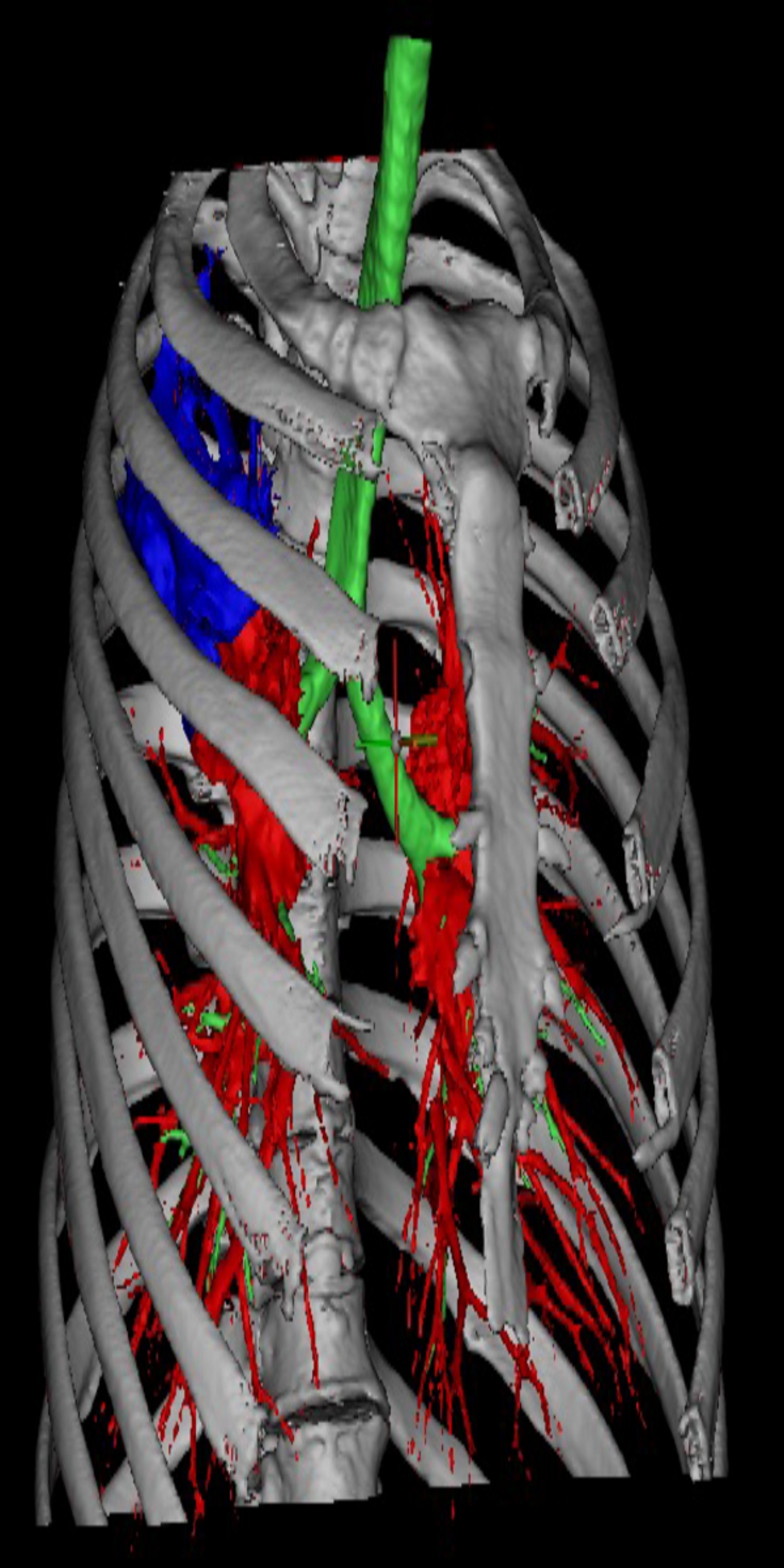
3D reconstruction of bronchial lung cancer space-occupying lesion model

**Fig. 3:**
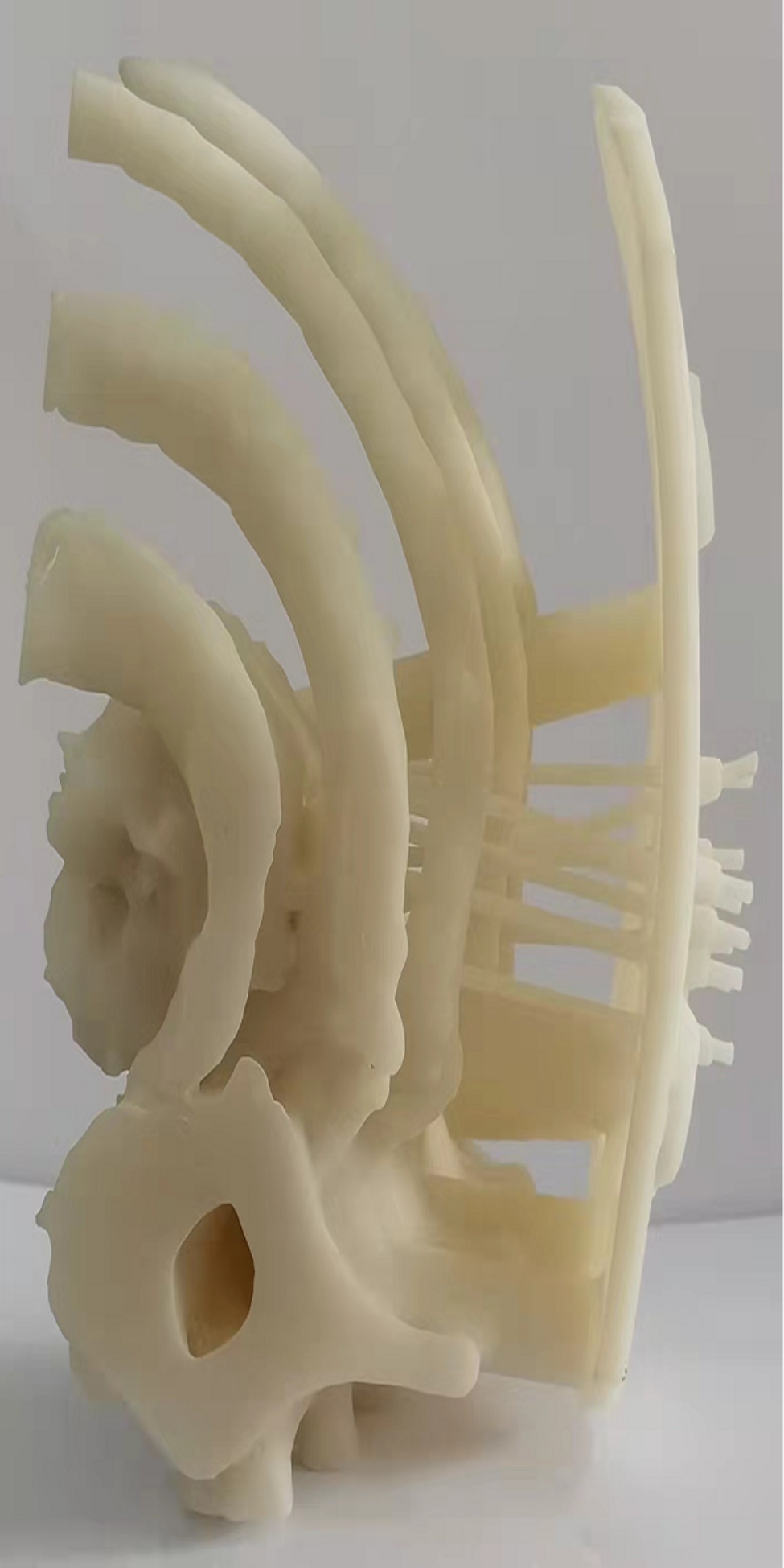
3D printed bronchial lung cancer space-occupying lesion model

Before class, the teachers provided 3D reconstructed bronchial lung cancer space-occupying lesion model of a real case to the group of students (Fig. 2), and also prepared lecture videos and supplementary materials for the course [10]. The students were given general diagnosis and treatment guidelines (Chinese and English versions), five reference papers related to the course’s topics [10]. Each student was required to review these materials in his/her own free time outside of class based on 3D printed model (Fig. 3), lecture videos, guidelines and reference papers. The class session was beginning with the instructor providing a brief introduction of the topic and the class agenda [10]. Next, a patient case with slides was presented. Then, check the patient at the bedside. After that, the students carried on small-group discussions under the instructor’s guidance [10]. During these discussions, the participants were encouraged to raise relevant questions and seek answers on the Internet and in the library database. Third, a student representative from each group gave a presentation to review the main points from the lesson, share their group’s answers to the questions posed, and discuss about any unsolved questions. Finally, the instructor summarized and went over the tough questions that were raised during discussion [10].

### Traditional group

Before the lecture, the students were instructed to preview the course, watching videos or reading materials in any extensive way [10]. These students were taught the equivalent content via the traditional teaching method; that is, the instructor provided a thorough explanation of the theoretical knowledge within the official framework. In other words, instructor teaching was the predominant approach [10].

### Assessment tools

According to previous studies [10, 33, 34], a pre-class and post-class quiz consisting of 20 questions, an examination after teaching in 2 weeks and a questionnaire with ten self-evaluation items were used to assess teaching effects.

### Theoretical assessment

For each group, before and after the classroom activities, the students were asked to complete a pre-class and post-class quiz consisting of 20 questions. All the questions were based on Bloom’s Taxonomy [34]. The pre-class quiz was the basic knowledge. The post-class quiz included the basic knowledge (18 questions) and the case analysis (2 questions). Questions in the two quizzes were different but same for every group. All students were also required to test their knowledge retention with an examination after teaching in 2 weeks. The main examination included the basic knowledge (14 questions) and the case analysis (6 questions) covering the aspects of bronchial lung cancer presented in the course, the question types are the single choice questions. 1 point for each question, and the total score is 20 points.

### Effectiveness assessment

Students were also asked to complete the questionnaire survey about their self-evaluation and satisfaction at the end of the teaching course. The questionnaire was modified and adopted by Oderinu [33] and Zhao [10]. The questionnaire with ten self-evaluation items involving clinical thinking ability, learning initiative, self-study ability, basic knowledge mastery, anatomical knowledge mastery, confidence in learning, ability to analyze and solve problems, comprehension of the knowledge, help to clinical thinking and level of satisfaction on the teaching method. Students graded the aspects based on a 5-point Likert scale, where each item was scored from 1 to 5 points, respectively [10, 33].

### Statistical analysis

All statistical analyses were carried using GraphPad prism 7 software. The measurement data were expressed as ${\rm{\bar X}}\,{\rm{ + }}\,{\rm{SD}}$. Data were assessed by independent sample t-test. The categorical data were analyzed by the chi-square test. P < 0.01 was considered statistically significant.

## Results

### Comparison of scores between the DPC and traditional groups

As shown in Table 2, as well as Supplementary Tables S1-3, we undertook a comparative analysis of the pre-class, post-class quiz scores and the main examination taken two weeks post-teaching in the DPC and traditional groups. The average pre-class quiz scores for the DPC group and traditional groups were 18.18 ± 1.03 and 18.44 ± 1.15, respectively, revealing no significant variance between the two groups in the pre-quiz (P > 0.01). Upon the completion of the class, the mean scores in the DPC and traditional groups were 17.87 ± 1.39 and 17.38 ± 1.35, respectively. An unpaired t test indicated a statistically significant disparity difference between the two groups’ post-quiz scores (P < 0.01), with the DPC group outperforming the traditional group. The mean scores for the main examination conducted two weeks after teaching were 16.82 ± 1.69 and 16.75 ± 1.59 in the DPC and traditional groups, respectively. No statistically significant difference was noted between the two groups in terms of the main examination (P > 0.05).

**Table 2:** Comparison of scores between the DPC and traditional groups

Item	DPC group (n = 211)	Traditional group (n = 211)	T	P value
Pre-class score	18.18 ± 1.03	18.44 ± 1.15	2.36	0.12
Post-class score	17.87 ± 1.39	17.38 ± 1.35	3.66	0.70
Score in 2 weeks	16.82 ± 1.69	16.75 ± 1.59	0.45	0.37

### Comparison of questionnaire survey scores between the DPC and traditional groups

Upon completion of the teaching course, a questionnaire survey, assessing self-evaluation and satisfaction was administered to the students. Our findings indicated that metrics such as clinical thinking ability, learning initiative, self-study ability, anatomical knowledge mastery, confidence in learning, ability to analyze and solve problems, comprehension of the knowledge, help to clinical thinking and level of satisfaction on the teaching method, all displayed significantly higher scores in the DPC group compared to the traditional group (P < 0.01) (Refer to Table 3 and Supplementary Table S4). However, no significant difference was observed between the two groups concerning mastery of basic knowledge (P = 0.02).

**Table 3:** Comparison of self-evaluation and satisfaction between the DPC and traditional groups

Item	DPC group (n = 211)	Traditional group (n = 211)	T	P value
Clinical thinking ability	4.62 ± 0.50	4.12 ± 0.76	3.11	< 0.01
Learning initiative	4.65 ± 0.49	4.26 ± 0.64	6.95	< 0.01
Self-study ability	4.59 ± 0.54	4.41 ± 0.67	3.14	< 0.01
Basic knowledge mastery	4.38 ± 0.55	4.25 ± 0.61	2.42	0.02
Anatomical knowledge mastery	4.58 ± 0.50	4.27 ± 0.62	5.66	< 0.01
Confidence in learning	4.49 ± 0.50	4.21 ± 0.58	5.39	< 0.01
Ability to analyze and solve problems	4.47 ± 0.61	4.29 ± 0.58	3.11	< 0.01
Comprehension of the knowledge	4.41 ± 0.69	4.02 ± 0.76	5.43	< 0.01
Help to clinical thinking	3.95 ± 0.78	3.64 ± 0.79	4.16	< 0.01
Satisfaction on the teaching method	4.32 ± 0.69	4.12 ± 0.70	3.00	< 0.01

### Comparison of the learning effect factors between the satisfied and unsatisfied DPC groups

In order to further evaluate the factors that influenced the participants’ learning experiences in the DPC group, we divided the DPC group into two sub-groups according to the questionnaire survey scores. Students who got scores greater than or equal to 3 points were assigned into the satisfied sub-group. As shown in Table 4, the notable difference between the satisfied and unsatisfied sub-groups was the comprehension of the knowledge. Within the satisfied sub-group, the average score for comprehension of the knowledge was 4.46 ± 0.68, higher than 4.07 ± 0.68 noted in the unsatisfied sub-group (P < 0.01). However, no significant differences were observed between the two groups regarding clinical thinking ability, learning initiative, self-study ability, basic knowledge mastery, anatomical knowledge mastery, confidence in learning, ability to analyze and solve problems, and help to clinical thinking (P > 0.05).

**Table 4:** Comparison of self-evaluation between the satisfied and unsatisfied DPC groups

Item	≤ 3 points (n = 27)	> 3 points (n = 184)	T	P value
Clinical thinking ability	4.48 ± 0.51	4.64 ± 0.49	1.51	0.13
Learning initiative	4.59 ± 0.50	4.66 ± 0.49	0.65	0.52
Self-study ability	4.59 ± 0.50	4.59 ± 0.55	< 0.01	0.10
Basic knowledge mastery	4.33 ± 0.48	4.39 ± 0.56	0.51	0.61
Anatomical knowledge mastery	4.59 ± 0.50	4.58 ± 0.50	0.16	0.87
Confidence in learning	4.48 ± 0.51	4.50 ± 0.50	0.13	0.90
Ability to analyze and solve problems	4.30 ± 0.54	4.50 ± 0.62	1.62	0.11
Comprehension of the knowledge	4.07 ± 0.68	4.46 ± 0.68	2.72	< 0.01
Help to clinical thinking	4.00 ± 0.83	3.95 ± 0.77	0.34	0.74

## Discussion

This study employed a combination of 3D printing technology with PBL and CBL teaching methods in respiratory diseases education, aiming to investigated their teaching efficacy. Prior research has demonstrated the divergence between PBL or CBL and traditional teaching method, underscoring the advantages of the former in establishing an authentic medical environment and promoting active, self-directed learning amongst students [35]. Given the intricate anatomical structure of the respiratory system, building its spatial representation necessitates a solid grounding in anatomical knowledge, and a strong aptitude for spatial understanding and thinking ability [36]. 3D printing bridges this gap, transforming 3D virtual objects into tangible physical entities, thus remedying the limitations and deficiencies inherent in traditional teaching tools [31]. The DPC and traditional groups comprised students with similar demographic and academic characteristics including gender, age, and performance at the start of the study (as shown in Table 1). These fourth-year students majoring in clinical medicine undertook a required course titled PBL designed to establish a connection between classroom instruction and clinical practice. Initial learning involved a series of lectures on all clinical specialties, complemented by laboratory skills sessions, eventually culminating in clinical immersion. Moreover, in this year, flexible and implementable teaching methods and teaching aids are allowed. Previous studies have focused on fourth-year students [10], junior students [32] or students enrolled in seven-year program [15]. Having acquired adequate basic knowledge through traditional classroom teaching during their early years of study [20, 21], the fourth-year students seemed to be ideal candidates for this study. All students underwent three theoretical assessments, including pre-class and post-class quizes and the main examination conducted two weeks post-teaching (as demonstrated in Fig. 1). Tan et al. [5] reported that superior theoretical scores in a group combining 3D printing with PBL group in teaching clinical nursing for congenital heart surgery, when compared to the traditional group. The amalgamation of PBL and CBL was found to augment teaching effectiveness in dental education [15]. Our results, in line with these earlier findings, showed that the DPC group outperformed the traditional group in the post-class quiz, suggesting the efficacy of the DPC approach in increasing theoretical test scores in the teaching of respiratory diseases, notably bronchial lung cancer. However, in terms of the main examination, the DPC group demonstrated scores comparable to those of the traditional group (as displayed in Table 2). This suggests that the DPC teaching method has no obvious advantages over the traditional teaching method in maintaining theoretical knowledge.

The outcomes of supplementary self-assessment questionnaire survey showed that the self-assessment scores of students in the DPC teaching method group were superior to those of the traditional teaching method group in terms of clinical thinking ability, learning initiative, self-study ability, anatomical knowledge mastery, confidence in learning, ability to analyze and solve problems, comprehension of the knowledge, help to clinical thinking and level of satisfaction on the teaching method (Table 3). These results suggest that DPC bolsters the pedagogical efficacy in respiratory disease education. This concurs with the findings of Zhao et at, who reported enhanced performance and clinical skills among medical students’ and residents’ via the integration of PBL and CBL in the clinical practical teaching of thyroid disease [10]. The combination of 3D printing technology with PBL achieved good results in teaching clinical nursing in congenital heart surgery [5]. Our results align with these studies, bridging the information gap concerning the efficacy of 3D printing, PBL and CBL in respiratory disease education, and demonstrating a comparable efficacy in teaching respiratory diseases. The DPC teaching method may yield gratifying self-learning experiences for students, thereby enhancing the pedagogical effectiveness. However, no statistically significant variance was discerned between the DPC and traditional groups in terms of basic knowledge mastery (Table 3). This might due to accumulation of adequate foundational knowledge by traditional classroom instruction during their junior studies [20, 21]. Considering that students majoring in clinical medicine are high-performing high school graduates, they exhibit robust capabilities to comprehend basic knowledge via traditional classroom instruction. Contrasting with the satisfied DPC sub-group, none of the aforementioned factors except for comprehension of the knowledge were identified as learning effect factors in the unsatisfied DPC sub-group. This implies that comprehension of the knowledge may be a crucial determinant of learning effectiveness and foundational to all the factors in the learning and teaching process. It may improve the ability to analyze and solve problems by integrating basic and clinical knowledge in combination with real clinical cases, and combining theory with practice.

## Conclusion

This study suggests that,DPC might be an efficacious strategy for enhancing senior medical students’ theoretical test scores and self-evaluation and satisfaction when learning about respiratory diseases. The DPC teaching methodology may prove beneficial in augmenting the pedagogical effectiveness, thus warranting wider adoption in teaching. However, the efficacy of DPC in educating students of other grades majoring in clinical medicine, or students majoring in other medical sciences, or for teaching other respiratory diseases beyond bronchial lung cancer remains to be comprehensively investigated. Future research should explore these avenues.

## Electronic supplementary material

Below is the link to the electronic supplementary material.

**Supplementary Material 1**. Pre-class quiz

**Supplementary Material 2**. Post-class quiz

**Supplementary Material 3**. Main examination

**Supplementary Material 4**. Questionnaire survey

## Data Availability

The datasets used and/or analyzed during the current study are available from the corresponding author on reasonable request.

## Abbreviations

PBL: Problem-Based Learning
CBL: Case-Based Learning
DPC: 3D printing technology with PBL and CBL
