# Supplementary material for: Enhancing medical education in respiratory diseases: efficacy of a 3D printing, problem-based, and case-based learning approach
Source: BMC Med Educ. 2023 Jul 17;23:512. doi: 10.1186/s12909-023-04508-6 (PMC10353117; doi:10.1186/s12909-023-04508-6)
Supplement: Supplementary file 1 — Supplementary Material 1. Pre-class quiz [file 12909_2023_4508_MOESM1_ESM.docx]

**Supplementary table 1 - Pre-class quiz**

入科考试

姓名： 学号： 年级：

1、属于下呼吸道的是：

A、口腔； B、鼻； C、咽； D、喉； E、气管

2、成年人喉介于：

A、第 2～5 颈椎之间；B、第 3～6 颈椎之间；C、第 2～7 颈椎之间；D、第 3～6 胸椎之间；E、第 3～7 胸椎之间

3、肺的构造和功能单位是

A、肺叶； B、肺泡； C、肺小叶； D、肺段； E、细支气管

4、以下哪项属于肺进展气体交换的场所

A、肺泡； B、肺叶支气管； C、肺段支气管； D、小支气管；E、细支气管

5、脏胸膜覆盖在：

A、心包的外表； B、肺外表； C、心脏外表； D、纵隔外表；E、膈肌外表

6、有关胸膜的描述，错误的选项是：

A、壁胸膜分为肋胸膜、膈胸膜、纵隔胸膜和胸膜顶四局部

B、左、右肺分别位于左、右胸膜腔内

C、左、右胸膜腔是两个完全封闭的腔，互不相通

D、胸膜腔内有少量浆液，呈负压

E、深吸气时，肺下缘也不能充满肋膈隐窝

7、壁胸膜的分部不包括：

A、肋胸膜； B、膈胸膜； C、纵隔胸膜； D、脏胸膜； E、胸膜顶

8、胸膜下界在锁骨中线相交于：

A、第 6肋； B、第 8肋； C、第 10 肋； D、第 11 肋； E、第 12 肋

9、纵隔边界中，错误的选项是：

A、前界为肋骨； B、后界为脊柱胸段； C、上达胸廓上口； D、向下至膈； E、两侧界为纵隔胸膜

10、不属于纵隔的器官是：

A、心包； B、心； C、胸主动脉； D、气管； E、肺

11、异物坠入下呼吸道时通常进入右肺， 与右主支气管特点有关。 关于右主支气管特点，正确的选项是：

A、粗、短、斜； B、粗、长、斜； C、粗、短、直； D、粗、长、直； E、细、短、直

12、支气管镜定位标志是：

A、环状软骨； B、气管隆嵴； C、气管杈； D、膜壁； E、以上都不是

13、吞咽时，关闭喉口的构造是：

A、甲状软骨； B、杓状软骨； C、环状软骨； D、会厌； E、气管软骨

14、正常肺下界的体表投影在腋中线处与第几肋相交：

A、第 6肋； B、第 7肋； C、第 8肋； D、第 9肋； E、第 10 肋

15、胸膜腔穿刺时，进针位置宜选：

A、腋中线 8-9 肋间，紧贴肋骨的下缘进针； B、腋中线 8-9 肋间，紧贴肋骨的上缘进针

C、腋中线 8-9 肋间，紧贴肋骨的中间进针； D、腋中线 6-7 肋间，紧贴肋骨的上缘进针

E、腋中线 6-8 肋间，紧贴肋骨的中间进针

16、以下哪些肌肉是不参与呼吸的肌肉：

A、膈肌； B、腹壁肌； C、肋间内肌； D、肋间外肌； E、胸锁乳突肌

17、气管切开部位选择正确的选项是：

A、第 1~3 气管软骨环； B、第 3~5 气管软骨环； C、第 4~6 气管软骨环； D、第 5~6 气管软骨环； E、第 6~7 气管软骨环

18、能够作为计数气管软骨环的标志是：

A、环状软骨弓； B、环状软骨板； C、杓状软骨； D、甲状软骨； E、会厌软骨

19、治疗急性感染性喉炎除控制感染外，还应同时应用下列何种药物减轻症状：

A、镇静剂； B、肾上腺皮质激素； C、止咳药； D、速尿； E、以上都不对

20、肺炎的心力衰竭常以什么衰竭为主

A、右心衰竭； B、左心衰竭； C、全心衰竭； D、末梢循环衰竭； E、以上都不对

Pre-class quiz

Name: Student number: Grade:

1、Which of the following belongs to the lower respiratory tract:

A. Oral cavity; B. Nose; C. Pharynx; D. Larynx; E. Trachea

2、Adult larynx is located at:

A. Between the 2nd and 5th cervical vertebrae;

B. Between the 3rd and 6th cervical vertebrae;

C. Between the 2nd and 7th cervical vertebrae;

D. Between the 3rd and 6th thoracic vertebrae; E. Between the third and seventh thoracic vertebrae

3、Which of the following is the structural and functional unit of the lung：

A. Lung lobe; B. Alveoli; C. Pulmonary lobules; D. Lung segment; E. Bronchiole

4、Which of the following is the site of lung progression gas exchange

A. Alveoli; B. Lobar bronchus; C. Segmental bronchus of lung; D. Bronchioles; E. Bronchiole

5、Visceral pleura covers:

A. Appearance of pericardium; B. Lung appearance; C. Appearance of heart; D. Mediastinal appearance; E. Diaphragmatic appearance

6、For the description of pleura, the wrong option is:

A. The parietal pleura is divided into costal pleura, diaphragmatic pleura, mediastinal pleura and pleural apex

B. The left and right lungs are located in the left and right pleural cavities respectively

C. The left and right pleural cavities are two completely closed cavities, which are not connected with each other

D. There is a small amount of serous fluid in the pleural cavity, showing negative pressure

E. During deep inhalation, the lower edge of the lung cannot fill the costophrenic recess

7、Segments of the parietal pleura do not include:

A. Costal pleura; B. Diaphragmatic pleura; C. Mediastinal pleura; D. Visceral pleura; E. Parietal pleura

8、The lower boundary of pleura intersects at the middle line of clavicle at:

A. The sixth rib; B. The 8th rib; C. The 10th rib; D. The 11th rib; E. 12th rib

9、The wrong option about mediastinum is:

A. The anterior boundary is the rib; B. The posterior boundary is the thoracic segment of the spine; C. Up to the upper opening of the thorax; D. Down to the diaphragm; E. Mediastinal pleura is defined on both sides

10、Which of the following is not a mediastinal organ:

A. Pericardium; B. Heart; C. Thoracic aorta; D. Trachea; E. Lungs

11、When the foreign object falls into the lower respiratory tract, it usually enters the right lung, which is related to the characteristics of the right main bronchus. For the characteristics of the right main bronchus, the correct option is:

A. Thick, short and oblique; B. Thick, long and oblique; C. Thick, short and straight; D. Thick, long and straight; E. Thin, short and straight

12、Which of the following is the bronchoscope positioning mark:

A. Cricoid cartilage; B. Tracheal crest; C. Tracheal bifurcation; D. Membrane wall; E. None of the above

13、When swallowing, the structure of closing the throat is:

A. Thyroid cartilage; B. Arytenoid cartilage; C. Cricoid cartilage; D. Epiglottis; E. Tracheal cartilage

14、The body surface projection of the lower boundary of the normal lung intersects the third rib at the axillary midline:

A. The sixth rib; B. The 7th rib; C. The 8th rib; D. The 9th rib; E. 10th rib

15、During pleural puncture, the needle entry position should be:

A. The axillary midline is 8-9 intercostals, and the needle is inserted close to the lower edge of the rib;

B. The axillary midline is 8-9 intercostals, and the needle is inserted close to the upper edge of the rib

C. The axillary midline is 8-9 intercostals, and the needle is inserted close to the middle of the rib;

D. The axillary midline is 6-7 intercostals, and the needle is inserted close to the upper edge of the rib

E. The axillary midline is 6-8 intercostals, and the needle is inserted close to the middle of the rib

16、Which of the following is a muscle that is not involved in breathing:

A. Diaphragm; B. Abdominal wall muscle; C. Intercostal muscles; D. External intercostal muscle; E. Sternocleidomastoid muscle

17、The correct choice of tracheotomy site is:

A. The 1st ~ 3rd tracheal cartilage ring; B. The 3rd ~ 5th tracheal cartilage ring; C. The 4th ~ 6th tracheal cartilage ring; D. The 5th ~ 6th tracheal cartilage ring; E. Tracheal cartilage ring 6 ~ 7

18、The signs that can be used to count the tracheal cartilage ring are:

A. Cricoid cartilage arch; B. Cricoid cartilage plate; C. Arytenoid cartilage; D. Thyroid cartilage; E. Epiglottic cartilage

19、In addition to controlling infection, which of the following drugs should be used to reduce symptoms in the treatment of acute infectious laryngitis:

A. Sedatives; B. Adrenocortical hormone; C. Cough medicine; D. Furosemide; E. None of the above is true

20、What kind of failure is often the main cause of heart failure in pneumonia

A. Right heart failure; B. Left heart failure; C. Total heart failure; D. Peripheral circulation failure; E. None of the above is true
