# Supplementary material for: Enhancing medical education in respiratory diseases: efficacy of a 3D printing, problem-based, and case-based learning approach
Source: BMC Med Educ. 2023 Jul 17;23:512. doi: 10.1186/s12909-023-04508-6 (PMC10353117; doi:10.1186/s12909-023-04508-6)
Supplement: Supplementary file 2 — Supplementary Material 2. Post-class quiz [file 12909_2023_4508_MOESM2_ESM.docx]

**Supplementary table 2 - Post-class quiz**

随堂考试

姓名： 学号： 年级：

1. 肺癌诊断金标准：

A、CT增强检查； B、组织病理学； C、PET-CT； D、MRI检查

1. 肺微小结节的定义：

A、直径5-10mm； B、直径≤5mm； C、直径＜3mm； D、直径≤10mm

1. 肺结节CT筛查的建议检查方式

A、低剂量CT检查； B、CT增强检查； C、MRI检查； D、DR检查

1. 以下哪些是肺结节恶性征像：

A、分叶、毛刺； B、空泡征； C、血管聚集征； D、以上都是

1. 低剂量CT的优势

A、肺部影响重叠少，密度分辨力高； B、相当于常规CT，低剂量CT扫描显著降低了X线辐射量； C、低剂量扫描可以减少管电流，降低CT球管出现过热的概率，延长其寿命，降低其成本； D、以上都是

1. 肺结节良性钙化

A、钙化占肺结节体积的10%以上； B、分层状、弥散性斑点状钙化； C、爆米花样或中心性大钙化； D、以上都是

1. 按密度分类，肺结节可分为：

A、实性结节； B、纯磨玻璃肺结节； C、混合性结节； D、以上都是

1. 肺结节在随访中出现哪项变化考虑恶性：

A、实性结节随访两年以上仍稳定； B、密度均匀或变淡； C、病灶稳定或增大，并出现实性成分； D、病灶迅速增大，倍增时间≤15天

9、LU-RADS肺结节CT报告系统关于肺结节描述的要素包括：

A、结节的位置、大小和密度； B、结节的形态、边缘以及与胸膜的关系； C、动态对比观察的变化； D、以上都是

10、肺结节分类为3L，随访复查的间隔时间是：

A、6-12周； B、3个月； C、半年； D、1年

11、肺癌最常见的转移途径是

A、血行转移； B、直接转移； C、支气管播散； D、淋巴道转移

12、在肺结节初始评估中，除影像学特征、危险因素外，还有哪些常用实验室指标可协助鉴别诊断：

A、高敏C反应蛋白； B、肿瘤标志物； C、T-SOPT； D、G试验

13、以下不是肺癌高危因素的是：

A、肺气肿； B、间质性肺病； C、遗传； D、职业或环境暴露

14、亚实性肺结节行PET-CT的指征是：

A、实性成分≤5mm； B、实性成分≤10mm； C、实性成分≥8mm； D、实性成分≥15mm

15、下列哪些选项提示肺结节恶性：

A、增大； B、稳定并密度增高； C、稳定或密度增高，并出现实性成分； D、缩小，但出现实性成分增大； E、出现形态学的恶性征象

16、以下哪种肺癌的放射治疗剂量最小：

A、鳞癌； B、腺癌； C、细支气管-肺泡癌； D、大细胞癌； E、小细胞癌

17、肺功能为评估肺癌患者能否耐受手术治疗的重要因素，一般要求患者术前肺功能的FVC超过多少？

A、2L； B、3L； C、4L； D、5L； E、1L

18、以下哪种肺癌的化疗疗效最好：

A、鳞癌； B、腺癌； C、细支气管-肺泡癌； D、大细胞癌； E、小细胞癌

19、肺结核球的特点以下哪项不正确：

A、多见于年轻患者； B、多位于肺上叶尖后段和下叶背段； C、病灶边界清楚，直径很少超过3cm； D、有时病灶内含有钙化点，周围有纤维结核灶； E、如有空洞形成，多为偏心空洞，洞壁不规则

20、下面哪项不符合细支气管-肺泡癌的表现：

A、两肺多有结节状播散病灶； B、病灶结节大小一致、分布均匀，密度较淡； C、结节病灶边界清楚，密度较高； D、病灶常呈进行性发展和增大； E、病人常有进行性呼吸困难

Post-class quiz

Name: Student number: Grade:

1、Gold standard for lung cancer diagnosis:

A. CT enhancement examination; B. Histopathology; C、PET-CT； D. MRI examination

2、Definition of mini pulmonary nodules:

A. 5-10mm in diameter; B. Diameter ≤ 5mm; C. Diameter ＜ 3mm; D. Diameter ≤ 10mm

3、Recommended examination methods for CT screening of pulmonary nodules

A. Low dose CT examination; B. CT enhancement examination; C. MRI examination; D. DR check

4、Which of the following are malignant signs of pulmonary nodules:

A. Split and burr; B. Vacuole sign; C. Vascular aggregation sign; D. All of the above

5、Advantages of low dose CT

A. The overlapping of lung effects is less, and the density resolution is high; B. Equivalent to conventional CT, low-dose CT scanning significantly reduces the amount of X-ray radiation; C. Low dose scanning can reduce the tube current, reduce the probability of overheating of CT tube, prolong its life and reduce its cost; D. All of the above

6、Benign calcification of pulmonary nodules

A. Calcification accounted for more than 10% of the volume of pulmonary nodules; B. Stratified and diffuse punctate calcification; C. Popcorn pattern or central large calcification; D. All of the above

7、According to density classification, pulmonary nodules can be divided into:

A. Solid nodule; B. Pure ground glass pulmonary nodules; C. Mixed nodules; D. All of the above

8、Which changes of pulmonary nodules during follow-up consider malignancy:

A. Solid nodules remained stable after more than two years of follow-up;

B. Uniform density or thinning;

C. The lesions were stable or enlarged with solid components;

D. The lesions increased rapidly, and the doubling time was ≤ 15 days

9、The elements of lung nodule description in LU-RADS lung nodule CT reporting system include:

A. Location, size and density of nodules;

B. The shape and edge of the nodule and its relationship with pleura;

C. Dynamic comparison of observed changes;

D. All of the above

10、Pulmonary nodules are classified as 3L. The interval between follow-up and reexamination is:

A. 6-12 weeks; B. 3 months; C. Half a year; D. 1 year

11、The most common way of metastasis of lung cancer is

A. Hematogenous metastasis; B. Direct transfer; C. Bronchial dissemination; D. Lymphatic metastasis

12、In the initial evaluation of pulmonary nodules, in addition to imaging features and risk factors, what common laboratory indicators can assist in differential diagnosis:

A. High sensitivity C-reactive protein; B. Tumor markers; C、T-SOPT； D. G test

13、Which of the following is not a high-risk factor for lung cancer

A. Emphysema; B. Interstitial lung disease; C. Heredity; D. Occupational or environmental exposure

14、The indications of PET-CT for sub solid pulmonary nodules are:

A. Solid composition ≤ 5mm; B. Solid component ≤ 10mm; C. Solid component ≥ 8mm; D. Solid component ≥ 15mm

15、Which of the following options suggest malignant pulmonary nodules:

A. Increase; B. Stable and high density; C. Stable or high density, with solid components; D. Reduced, but the solid component increased; E. Appearance of morphological malignant signs

16、Which of the following lung cancer has the lowest radiation dose

A. Squamous cell carcinoma; B. Adenocarcinoma; C. Bronchioloalveolar carcinoma; D. Large cell carcinoma; E. Small cell carcinoma

17、Lung function is an important factor to evaluate whether patients with lung cancer can tolerate surgical treatment. How much FVC of preoperative lung function is generally required?

A、2L； B、3L； C、4L； D、5L； E、1L

18、Which of the following is the most effective chemotherapy for lung cancer:

A. Squamous cell carcinoma; B. Adenocarcinoma; C. Bronchioloalveolar carcinoma; D. Large cell carcinoma; E. Small cell carcinoma

19、Which of the following features of tuberculoma is incorrect:

A. More common in young patients;

B. Most of them were located in the posterior segment of the upper lobe tip and the dorsal segment of the lower lobe;

C. The boundary of the lesion was clear, and the diameter was rarely more than 3cm; D. Sometimes there are calcifications in the focus and fibrous tuberculosis around it;

E. If any cavity is formed, it is mostly eccentric cavity and the tunnel wall is irregular

20、Which of the following is not consistent with the appearance of bronchioloalveolar carcinoma:

A. There were nodular disseminated lesions in both lungs;

B. The size and distribution of lesions and nodules were uniform and the density was light;

C. The boundary of nodules was clear and the density was high;

D. The lesions often showed progressive development and enlargement;

E. Patients often have progressive dyspnea
