# Supplementary material for: Enhancing medical education in respiratory diseases: efficacy of a 3D printing, problem-based, and case-based learning approach
Source: BMC Med Educ. 2023 Jul 17;23:512. doi: 10.1186/s12909-023-04508-6 (PMC10353117; doi:10.1186/s12909-023-04508-6)
Supplement: Supplementary file 3 — Supplementary Material 3. Main examination [file 12909_2023_4508_MOESM3_ESM.docx]

**Supplementary table 3 -Main examination**

出科考试

姓名： 学号： 年级：

1. 下列哪项不是周围型肺癌的特点：

A、易误诊为炎症和结核； B、组织学以腺癌为多见； C、X线上阴影呈圆形或类圆形； D、易形成厚壁空洞，但空洞不伴有液平； E、易侵犯胸膜引起恶性胸腔积液

2、戒烟使患肺癌的危险性随戒烟年份的延长而逐渐降低，戒烟多少时间以上患肺癌的风险性才与不吸烟者相近？

A、5年； B、10年； C、15年； D、20年；E、25年

3、下面哪种类型为小细胞癌：

A、燕麦细胞癌； B、类癌； C、透明细胞癌； D、巨细胞癌；E、细支气管-肺泡细胞癌

4、癌性阻塞性肺炎的特点：

A、起病急骤，先有寒战、高热； B、有咳嗽、咳痰等呼吸道症状； C、抗菌素治疗效果好； D、病灶吸收迅速而完整；E、病灶吸收后出现块状阴影

5、下面哪种类型肺癌的治疗以化疗为主，辅以手术和（或）放疗：

A、鳞癌； B、腺癌； C、大细胞癌； D、小细胞肺癌； E、细支气管-肺泡癌

6、某男，56岁，因反复咳嗽，咳痰13年，痰中带血2周入院，曾因咳嗽，咳痰多次住院，明确诊断为COPD。有30多年的吸烟史。每日20支以上。入院查体：气管居中，桶状胸，肋间隙增宽，双肺叩诊呈过清音，双肺呼吸音减低。双肺可闻及细小湿啰音，胸部射线提示双肺纹理增多、紊乱，肺透光度增强，双膈低平，右肺门阴影增宽增大。目前此患者最可能的诊断是:

A、右肺癌。； B、肺门淋巴结结核； C、右肺炎； D、COPD； E、右肺中叶不张

7、容易变性坏死，形成空洞的肺癌是：

A、小细胞肺癌； B、肺泡细胞癌； C、腺癌； D、大细胞肺癌； E、鳞癌

8、诊断肺癌最可靠的手段是：

A、病史及体征； B、胸部射线检查； C、细胞学和气管镜检查； D、胸部磁共振检查； E、放射性核素检查

9、非小细胞肺癌痰中找到癌细胞的阳性率一般为：

A、50-60%； B、60-70%； C、70-80%； D、80-90%； E、100%

10、患者男性，60岁。声音嘶哑、低热、咳嗽、咳痰三个月。有结核接触史。双肺无阳性体征，胸片是提示：左上肺见直径3cm、密度较高的球形病灶。最可能的诊断是：

A、周围型肺癌； B、结核球； C、炎性假瘤； D、肺囊肿； E、机化性肺炎

11、癌性空洞的线典型表现为：

A、薄壁空洞，内壁光滑； B、厚壁空洞，内壁凹凸不平； C、厚壁空洞，有液平； D、薄壁空洞，周围有卫星灶； E、厚壁空洞，外壁模糊

12、患者男性，55岁。咳嗽、持续痰中带血2个月，吸烟指数500支/年，无发热及咳脓痰病史。查体发现储藏者明显。可能的诊断是：

A、支气管扩张； B、浸润型肺结核； C、支气管肺癌； D、肺脓肿

13、患者男性，65岁。母亲患肺腺癌已去世。吸烟指数400支/年.体检胸部低剂量CT示右上肺8mm结节；下一步最佳方案是：

A、外科手术，切除病灶； B、完善肿瘤指标； C、完善胸高分辨CT+靶扫+三维重建； D、气管镜检查；

14、以下哪项不是肺结节恶性征像：

A、分叶、毛刺； B、空泡征； C、血管聚集征； D、边界清楚，纯磨玻璃结节

15、肺结节在随访中出现哪项变化需要警惕及进一步处理：

A、病灶迅速增大，有血管穿行，内有空泡； B、密度均匀或变淡； C、病灶稳定或增大，并出现实性成分； D、实性结节随访两年以上仍稳定

16、患者男性，22岁，近期熬夜，消瘦，乏力。胸X线提示双肺多发结节，可能的诊断是：

A、支气管肺泡细胞癌； B、肺结核； C、支原体肺炎； D、泛细支气管炎

17、患者女性，58岁，2年前左肺腺癌手术，Ia期，术后常规复查随访。此次为1个月前复查胸低剂量CT发现左下肺9mm结节。下一步最佳处理方案为：

A、完善肿瘤指标和完善胸高分辨CT+靶扫+三维重建； B、气管镜检查； C、外科手术，切除病灶； C、肺穿检查

18、肺癌伴癌综合征不包括哪项：

A、Cushing综合征； B、肥大性骨关节病； C、类癌综合征； D、Horner综合征

19、肺癌痰脱落细胞学阳性率，与下列哪项无关：

A、标本是会否符合要求； B、病理科医生水平； C、肺癌的组织病理学类型； D、肿瘤生长部位； E、患者年龄

20、40岁以上的长期吸烟者以下哪些情况要考虑肺癌的可能性：

A、无明显诱因的刺激性咳嗽2-3周，治疗无效；

B、原因不明的四肢关节疼痛及杵状指（趾）；

C、原有慢性呼吸道疾病的咳嗽性质改变；

D、无中毒症状的进行性增加的血性胸腔积液；

E、以上均是

Examination (after teaching in 2 weeks)

Name: Student number: Grade:

1、Which of the following is not a characteristic of peripheral lung cancer:

A. Easily misdiagnosed as inflammation and tuberculosis;

B. Adenocarcinoma was the most common histology;

C. The shadow on X-ray is round or quasi round;

D. It is easy to form thick wall cavity, but the cavity is not accompanied by liquid level;

E. Easy to invade pleura and cause malignant pleural effusion

2、Smoking cessation makes the risk of lung cancer gradually decrease with the extension of smoking cessation years. How long does it take to quit smoking before the risk of lung cancer is similar to that of non-smokers?

A. 5 years; B. 10 years; C. 15 years; D. 20 years; E. 25 years

3、Which of the following types is small cell carcinoma:

A. Oat cell carcinoma; B. Carcinoid; C. Clear cell carcinoma; D. Giant cell carcinoma; E. Bronchioloalveolar cell carcinoma

4、Which one is the characteristics of cancerous obstructive pneumonia:

A. The onset is sudden, with chills and high fever first;

B. Have respiratory symptoms such as cough and expectoration;

C. The therapeutic effect of antibiotics is good;

D. The lesions were absorbed rapidly and completely;

E. Massive shadow appeared after lesion absorption

5、Which of the following types of lung cancer is mainly treated with chemotherapy, supplemented by surgery and / or radiotherapy:

A. Squamous cell carcinoma; B. Adenocarcinoma; C. Large cell carcinoma; D. Small cell lung cancer; E. Bronchioloalveolar carcinoma

6、A 56-year-old male was hospitalized for repeated cough and expectoration for 13 years with blood in sputum for 2 weeks. He was hospitalized for many times due to cough and expectoration, and was definitely diagnosed as COPD. He has a smoking history of more than 30 years. More than 20 per day. Admission physical examination: the trachea is in the middle, the barrel chest, the costal space is widened, the percussion of both lungs is over clear, and the respiratory sound of both lungs is reduced. Small wet rales can be heard in both lungs. Chest rays suggest that the markings of both lungs are increased and disordered, the lung transmittance is enhanced, the diaphragms are low and flat, and the shadow of the right hilar is widened and enlarged. At present, the most likely diagnosis of this patient is:

A. Right lung cancer.; B. Hilar lymph node tuberculosis; C. Right pneumonia; D、COPD； E. Right middle lobe atelectasis

7、Which of the following is a lung cancer prone to degeneration, necrosis and cavity formation:

A. Small cell lung cancer; B. Alveolar cell carcinoma; C. Adenocarcinoma; D. Large cell lung cancer; E. Squamous cell carcinoma

8、Which of the following is the most reliable means to diagnose lung cancer:

A. Medical history and signs; B. Chest radiography; C. Cytology and tracheoscopy; D. Chest magnetic resonance examination; E. Radionuclide examination

9、The positive rate of cancer cells found in sputum of non-small cell lung cancer is generally:

A、50-60%； B、60-70%； C、70-80%； D、80-90%； E、100%

10、The patient was male, 60 years old. Hoarseness, low fever, cough and expectoration for three months. Have a history of tuberculosis exposure. There were no positive signs in both lungs. The chest X-ray showed that there were spherical lesions with a diameter of 3cm and high density in the left upper lung. The most likely diagnosis is:

A. Peripheral lung cancer; B. Tuberculoma; C. Inflammatory pseudotumor; D. Pulmonary cyst; E. Organic pneumonia

11、Which of the following is a typical manifestation of cancerous cavity:

A. Thin wall cavity, smooth inner wall; B. Thick wall cavity and uneven inner wall; C. Thick wall cavity with liquid level; D. Thin wall cavity with satellite focus around; E. Thick wall cavity, fuzzy outer wall

12、The patient was male, 55 years old. Cough, blood in sputum for 2 months, smoking index of 500 cigarettes/year, no history of fever and expectoration. Physical examination found that the storer was obvious. Which is a possible diagnosis:

A. Bronchiectasis; B. Invasive pulmonary tuberculosis; C. Bronchial lung cancer; D. Lung abscess

13、The patient was male, 65 years old. Her mother died of lung adenocarcinoma. The smoking index is 400 cigarettes/year. On the regular physical examination, chest low-dose CT showed an 8mm nodule in the right upper lung; The next best solution is:

A. Surgery, resection of lesions; B. Tumor indicators; C. Chest high-resolution CT + target scan + three-dimensional reconstruction; D. Tracheoscopy

14、Which of the following is not a malignant sign of pulmonary nodules:

A. Burr and split leaf; B. Vacuole sign; C. Vascular aggregation sign; D. Clear boundary, pure ground glass nodule

15、Which changes of pulmonary nodules need vigilance and further treatment during follow-up:

A. The focus increased rapidly, with blood vessels passing through and vacuoles in it; B. Uniform density or thinning; C. The lesions were stable or enlarged with solid components; D. Solid nodules remained stable after more than two years of follow-up

16、The patient is a 22-year-old male, who recently stayed up late, thin and weak. Chest X-ray shows multiple nodules in both lungs. The possible diagnosis is:

A. Bronchoalveolar cell carcinoma; B. Tuberculosis; C. Mycoplasma pneumonia; D. Panbronchiolitis

17、The patient, a 58-year-old female, underwent surgery for left lung adenocarcinoma two years ago, stage IA, and underwent routine follow-up. This time, a 9 mm nodule in the left lower lung was found by chest low-dose CT one month ago. The next best solution is:

A. Tumor indicators and chest high-resolution CT + target scan + three-dimensional reconstruction; B. Tracheoscopy; C. Surgery, resection of lesions; C. Lung puncture examination

18、Lung cancer with cancer syndrome does not include:

A. Cushing syndrome; B. Hypertrophic osteoarthropathy; C. Carcinoid syndrome; D. Horner syndrome

19、The positive rate of sputum exfoliative cytology in lung cancer has nothing to do with which of the following:

A. Whether the specimen will meet the requirements; B. Level with pathologists; C. The histopathological type of lung cancer; D. Tumor growth site; E. Patient age

20、For long-term smokers over the age of 40, which of the following situations should consider the possibility of lung cancer:

A. Irritant cough without obvious inducement for 2-3 weeks, and the treatment is ineffective;

B. Unexplained limb joint pain and clubbing fingers (toes);

C. Change of cough nature of original chronic respiratory diseases;

D. Progressive increased bloody pleural effusion without toxic symptoms;

E. All of the above
