# Supplementary material for: Enhancing medical education in respiratory diseases: efficacy of a 3D printing, problem-based, and case-based learning approach
Source: BMC Med Educ. 2023 Jul 17;23:512. doi: 10.1186/s12909-023-04508-6 (PMC10353117; doi:10.1186/s12909-023-04508-6)
Supplement: Supplementary file 4 — Supplementary Material 4. Questionnaire survey [file 12909_2023_4508_MOESM4_ESM.docx]

**Supplementary table 4 - Questionnaire survey**

问卷调查表

姓名： 学号： 年级：

评估（1-5，1最差，5最好）

1. 是否提高临床思维能力 1 2 3 4 5
2. 是否提高学习积极性 1 2 3 4 5
3. 是否提高自学能力 1 2 3 4 5
4. 是否提高基础知识的掌握 1 2 3 4 5
5. 是否提高分析和解决问题的能力 1 2 3 4 5
6. 是否提高学习肺占位性病变的兴趣 1 2 3 4 5
7. 是否提高对肺占位性病变的解剖学知识的认知 1 2 3 4 5
8. 是否提高对肺占位性病变的知识的理解能力 1 2 3 4 5
9. 是否对临床教学有帮助吗 1 2 3 4 5
10. 是否对教学方法满意吗 1 2 3 4 5

Questionnaire

Name: Student number: Grade:

Assessment (1-5, 1 worst, 5 best)

1. Can you improve your clinical thinking ability? 1 2 3 4 5
2. Can you increase your learning initiative? 1 2 3 4 5
3. Can you improve your self-study ability? 1 2 3 4 5
4. Can you improve basic knowledge mastery? 1 2 3 4 5
5. Can you improve your ability to analyze and solve problems?

1 2 3 4 5

1. Can you increase your interest in learning about lung space-occupying lesion?

1 2 3 4 5

1. Can you improve your understanding of anatomical knowledge of lung space-occupying lesion?

1 2 3 4 5

1. Can you improve your comprehension of the knowledge of lung space-occupying lesion?

1 2 3 4 5

1. Do you think the teaching method is help to clinical teaching?

1 2 3 4 5

1. How is the level of satisfaction on the teaching method?

1 2 3 4 5
